# Long-Term Immunogenicity Studies of Formalin-Inactivated Enterovirus 71 Whole-Virion Vaccine in Macaques

**DOI:** 10.1371/journal.pone.0106756

**Published:** 2014-09-08

**Authors:** Chia-Chyi Liu, Chyi-Sing Hwang, Wun-Syue Yang, Dan-Chin Tsai, Sze-Hsien Wu, Ai-Hsiang Chou, Yen-Hung Chow, Suh-Chin Wu, Jen-Ren Wang, Jen-Ron Chiang, Chin-Cheng Huang, Chien-Hsiung Pan, Pele Chong

**Affiliations:** 1 National Institute of Infectious Diseases and Vaccinology, National Health Research Institutes, Zhunan Town, Miaoli County, Taiwan; 2 Animal Health Research Institute, Council of Agriculture, Tamsui, Taipei, Taiwan; 3 Institute of Biotechnology, Department of Life Science, National Tsing Hua University, Hsinchu, Taiwan; 4 Centers for Disease Control, Taipei, Taiwan; 5 Graduate Institute of Immunology, China Medical University, Taichung, Taiwan; University of Massachusetts Medical Center, United States of America

## Abstract

Enterovirus 71 (EV71) has caused epidemics of hand, foot and mouth diseases in Asia during the past decades and no vaccine is available. A formalin-inactivated EV71 candidate vaccine (EV71vac) based on B4 subgenotype has previously been developed and found to elicit strong neutralizing antibody responses in mice and humans. In this study, we evaluated the long-term immunogenicity and safety of this EV71vac in a non-human primate model. Juvenile macaques were immunized at 0, 3 and 6 weeks either with 10 or 5 µg doses of EV71vac formulated with AlPO_4_ adjuvant, or PBS as control. During the 56 weeks of studies, no fever nor local redness and swelling at sites of injections was observed in the immunized macaques. After single immunization, 100% seroconversion based on 4-fold increased in neutralization titer (Nt) was detected in EV71vac immunized monkeys but not PBS controls. A dose-dependent IgG antibody response was observed in monkeys receiving EV71vac immunization. The Nt of EV71vac immunized macaques had reached the peak after 3 vaccinations, then decreased gradually; however, the GMT of neutralizing antibody in the EV71vac immunized macaques were still above 100 at the end of the study. Correspondingly, both dose- and time-dependent interferon-γ and CD4^+^ T cell responses were detected in monkeys receiving EV71vac. Interestingly, similar to human responses, the dominant T cell epitopes of macaques were identified mainly in VP2 and VP3 regions. In addition, strong cross-neutralizing antibodies against most EV71 subgenotypes except some C2 and C4b strains, and Coxsackievirus A16 were observed. In summary, our results indicate that EV71vac elicits dose-dependent T-cell and antibody responses in macaques that could be a good animal model for evaluating the long-term immune responses elicited by EV71 vaccines.

## Introduction

Enterovirus 71 (EV71), a non-enveloped RNA virus of the family *Picornaviridae*, genus *Enterovirus* was first identified in California 45 years ago and subsequently reported in many parts of world [1], [2]. EV71 and Coxsackievirus A16 (CVA16) are two major enteroviruses that cause epidemics of hand-foot-and-mouth disease (HFMD), but EV71 infection is associated with severe neurological diseases in young children [2]. Based on the sequence of the VP1 gene, EV71 is currently classified into 3 genotypes A, B and C, and genotypes B and C are further divided into B1–B5 and C1–C5 subgenotypes [2]. Genetic mutation and recombination between the RNA genome are known to contribute to the evolution of enterovirus [3], [4]. The mutation rate in enterovirus is estimated as high as one mutation per neosynthesized genome [5]. Evidences of the intratypic and intertypic recombination in enterovirus have been reported during the recent epidemic in Asia. For example, the intertypic recombination of genes derived from EV71 and other enteroviruses such as CVA16, 14 and 4 had been happened to the emergence of subgenotype C4a and C4b [6]. The same phenomenon was also reported in subgenotype B and C2 [7], [8]. The recombination process could allow EV71 to escape the host immunity and cause epidemic. EV71 outbreaks have occurred in the Asia-Pacific areas and caused many deaths in Taiwan, mainland China and Vietnam [1], [2], [9]. Unfortunately, neither a prophylactic vaccine nor antiviral therapy against HFMD is available now.

EV71 has an icosahedral viral particle containing a single, positive-sense RNA (7.5–8.5 kb) and four structural capsid proteins, including VP1, VP2 and VP3 on the external surface of the virion and VP4 within the interior of the viral particle [10]. Similar to other enteroviruses, the VP1, VP2 and VP3 of EV71 are responsible for the induction of host immunity, but VP1 has been reported to contain the major neutralization epitopes [11]. Evidence from studies in mice and humans indicated that T cell immunity played a critical role in control of the disease and inhibition of virus replication. A decrease in cellular immunity or interferon (IFN)-γ production is correlated with more severe clinical outcomes of EV71 infection, whereas neutralizing antibody titers show no differences between different EV71-realad diseases outcomes [12], [13]. In contrast, neutralizing antibodies are important for preventing EV71 infection and blocking of EV71 transmission. It has been reported that higher transmission rate was found to be correlated with a lower prevalence of neutralizing antibodies in children [14]–[15]. Mice were fully protected against EV71 infections by the passive transfer of neutralizing antibodies [16]. An ideal EV71 vaccine should be capable of inducing both cellular immunity and neutralizing antibodies against virus infection.

Several types of EV71 vaccine candidates, including inactivated virus vaccine, attenuated virus vaccine [17], subunit/peptide-based vaccine [16], [18] and virus-like particle vaccine [19], have been developed. The inactivated EV71 whole-virion candidate vaccines are the most promising and are currently in clinical trials [20]–[24]. Although these clinical trials have reported that the vaccine is safe and immunogenic, the long-term safety and immunogenicity of these EV71 vaccine candidates are unclear. Therefore, we used macaques (non-human primate) as a model to evaluate the safety and long-term (over one year) immunogenicity of a formalin-inactivated EV71 whole-virion vaccine candidate (EV71vac) in this study. Our data demonstrated that EV71vac induced strong T- and B-cell immune responses and high cross-neutralizing antibodies against several EV71 subgenotypes but not CVA16. Therefore, these results provide valuable information for future HFMD vaccine development.

## Materials and Methods

### Ethics Statement

All experiments were conducted in accordance with NHRI Laboratory Animal Center guidelines and approved by the NHRI Institutional Animal Care and Use Committee (Approval No. NHRI-IACUC-098033-A & NHRI-IACUC-099053-A). An US patent (US20120045468 A1) has been filed with the results presented in this study.

### Animal welfare and steps taken to ameliorate suffering

Ten young (1–3 years old) Taiwan macaques (*Macaca cyclopis*) using in this study were obtained from the Animal Health Research Institute of Taiwan, and pre-screened for no anti-EV71 IgG antibody (titer <200) and neutralizing antibody (Nt <8). Animals were housed in a secured SPF room with locked double-doors and cameras to monitor the animal activity. Fresh fruit with fixed-formula diets (∼4% of the body weight) on the basis of two times a day are used to feed the monkeys. At the beginning of this study, monkeys are moved into individual cage (62×62×72 cm) with the double-door, squeeze-back design to allow the monkeys to contact each other in vision but avoiding physical touch. To enrich the environment, some toys and different fresh fruit are provided to the animals. If an animal appears to be in poor condition, an adequate medical treatment or environment improvement will be used to relieve the pain or distress based on the recommendation of certificated veterinarians. All monkeys are moved back to the community cages with bigger space and better interaction with other monkeys in the end of this study.

Animals were chemically restrained with Zoletil (1.4–4.5 mg/Kg) before immunization or bleeding. The vital sign was monitored by breath and heart beating during the anesthesia. For bleeding, about 5 ml of heparinized blood was drawn from femoral veins each 3 weeks for the first 3 months, then every 3 months until the end of this study. After drawn, monkeys were placed back into original cages for recovery, which usually needs 15∼20 minutes and under the veterinarian's supervision. A humane euthanasia is performed by pentobarbital intravenous injection (100 mg/Kg) under the certificated veterinarian's recommendation, if animal is out of control such as unknown fever, severe diarrhea (containing blood, mucus and pus), moribund or not eating/drinking and doesn't show improvement following medication or environmental enrichment.

### Monkey immunization

For immunization, monkeys were randomly divided into three groups. The high dose group (A16, A25, A28 and A31) was vaccinated by intramuscular (im) injection with 10 µg of EV71vac formulated with AlPO_4_ (alum). The low dose group (A11, A14, A18 and A27) was im vaccinated with 5 µg of EV71vac formulated with alum. The control group (A20 and A21) was im vaccinated with PBS plus 300 µg alum. All groups received two boosts with the same vaccine and dose three and six weeks later.

### Cells and viruses

All EV71 strains used for the in vitro neutralization assay were grown in Vero cells. Vero cells were kindly provided by the Taiwan Centers of Disease Control (Taiwan CDC). The EV71/E59 strain (B4 subgenotype), a clinical isolate of the EV71 virus, and C4D (C4a subgenotype) were obtained from the Taiwan CDC. EV71 clinical isolates 0204/TW86 (B1 subgenotype), N0692/TW08 (B5 subgenotype), N0448/TW08 (B5 subgenotype), 5746/TW98 (C2), 4643/TW98 (C2), 1757/TW98 (C4a), N3340/TW02 (C4b), and Coxsackievirus A16 5079/TW98 were obtained from Nation Cheng Kung University, Taiwan.

### EV71 vaccine candidate (EV71vac)

The inactivated EV71vac vaccine made from EV71/E59 strain (B4 subgenotype) was produced and fully characterized as previously reported [25]. The EV71vac products (20 µg of vaccine bulk formulated with 3 mg of AlPO_4_ in 1 mL of PBS) were stored at 4°C for the animal study.

### ELISA

Specific IgG titer against EV71/E59 was determined by ELISA [22]. Inactivated EV71/E59 (1 µg/mL) was coated onto 96-well microplates (NUNC) overnight. After being washed with 0.5% Tween 20 in PBS (PBST) and blocked with 1% bovine serum albumin (BSA)/PBS at room temperature (RT), 100 µL of two-fold serially diluted sera (beginning with 1∶200) were added to the microplates. The microplates were incubated at RT for 2 hours, washed with PBST and 100 µL of goat anti-monkey IgG antibody conjugated with HRP (1∶5000; AbD Serotec) was added for 1 hour at RT. After washing, 100 µL of chromogenic substrate was added and allowed to incubate for 20 minutes. Absorbance was measured at 405 nm using a spectrophotometer. The IgG titer was defined as the endpoint of serial dilution with optical density two-folds higher than the background.

### Virus neutralizing assay

Sera in a two-fold serial dilution with cell culture medium were added to microtubes. A suspension of 200 TCID_50_ of virus (400 µL) was added to a tube containing diluted sera (400 µL). After incubation at 4°C for 18–24 hours, 100 µL of virus-serum mixture was added to Vero cells cultured in 96-well plates and incubated for seven days at 37°C, and TCID_50_ values were measured by counting cytopathic effects. The 50% neutralization inhibition dose was obtained by calculating the geometric reciprocal of the serum dilution yielding 50% reduction using the Reed-Muench method.

### ELISPOT assay

Multiscreen plates (Millipore) were coated with anti-human IFN-γ antibody (2 µg/ml) or anti-human IL-4 antibody (5 µg/mL; all from BD). After washing and blocking with culture medium, duplications of 5×10^5^ fresh PBMCs were added with concanavalin A (5 µg/mL; Sigma), inactivated EV71/E59 (50 µg/ml) or 5 µg/mL of VP1, VP2 or VP3 peptide mixture prepared from a series of 15-mer peptides with 10-mer overlapping to cover whole VP1 (1–295 aa), VP2 (1–254 aa) or VP3 (1–242 aa), respectively [20], [24]. After 40 hours of incubation, the plates were washed and incubated with a biotinylated antibody against IFN-γ (1 µg/mL) or IL-4 (2 µg/mL) for 2 hours at 37°C. HRP-conjugated avidin was added and incubated for 1 hour at 37°C. The assays were developed with DAB (Invitrogen) and stopped with tap water. Data were analyzed using ImmunoSpot software (CTL) and presented as the number of spot-forming cells (SFCs)/10^6^ PBMCs.

### Intracellular cytokine staining

Fresh PBMCs (2×10^6^) from monkeys that received EV71vac immunization twice (week 6) were cultured in 96-well round-bottomed plates at 37°C in a 5% CO2 incubator and stimulated with inactivated EV71, PMA plus ionomycin or medium alone. Cytokines were retained in the cytoplasm by adding Golgi-plug and Golgi-stop inhibitors 4 hours after stimulation. After 18 hours of incubation, cells were harvested, washed with staining buffer (PBS with 0.5% BSA) and blocked with anti-CD16 and anti-CD32 antibodies for 20 min at 4°C. The surface markers were stained with anti-monkey CD3, CD4 and CD95 conjugated with APC, PerCP and PE, respectively (all from BD) for 20 min at 4°C. After washing, cells were permeabilized with Cytofix buffer (BD Bioscience) and stained with FITC-conjugated anti-monkey TNF for 20 min at 4°C. Finally, cells were resuspended in 0.5 ml fixing solution (eBioscience) and assayed by FACS Caliber. Data were analyzed by FlowJo software (Treestar), and the CD3^+^ CD4^+^ T cell population was gated and presented.

### Statistical analysis

All statistical analyses were performed using two-way ANOVA with Bonferroni post-tests from Prism 5.0 software (GraphPad), if there is no additional description.

## Results

### Vaccine safety

Ten juvenile macaque monkeys (1–3 years old) with a body weight range between 1.7–5.0 kg (Median: 2.8 kg) were divided into three groups, and every group had an equal number of male and female monkeys. Two groups (4 macaques/group) were vaccinated three times at 0, 3 and 6 weeks either with high (10 µg) or low (5 µg) doses of EV71vac; and the control PBS group (two macaques) was immunized with PBS/alum. Physiological conditions and adverse side effects were monitored by daily routine examination. During the 56 weeks of the study, no vaccine-related side effect such as fever, local specific redness or swelling at the site of injections was observed in the immunized monkeys (Table S1). The full blood count analysis were also shown to be homeostatic status in RBC, platelet and eosinophil count; only WBC count was found to be varied during the period of the experiment (Table S2). The current safety data of EV71vac from the immunized monkeys was found to be consistent with the results obtained from the previous human clinical trial [22].

### Specific anti-EV71 IgG antibody responses

After a single immunization, the EV71vac regardless dosage elicited 100% seroconversion (a 4-fold increase in IgG antibody titer) in the immunized monkeys as shown in Table 1. In contrast, the PBS control group after immunization both anti-EV71 neutralization titer and IgG titer remained to be <8 and <200, respectively. The geometric mean titer (GMT) of specific anti-EV71 IgG in high- and low-dose immunized monkeys was found to be 1903 and 1600, respectively. The GMT of anti-EV71 IgG in EV71vac immunized monkeys increased further following the second and the third doses and reached a peak at week 12. After 2 doses the high- dose immunized monkeys had significantly higher anti-EV71 IgG titers than those monkeys immunized with low-dose (*p*<0.05). Anti-EV71 IgG titers were found to decline quickly after the peak but still remained detectable at 56 weeks, the end of the experiment. The GMT of anti-EV71 IgG at week 56 was tested to be 476 and 283 for high- and low-dose immunized monkey groups, respectively. The current results suggest that EV71vac could elicit strong and quick anti-EV71 IgG antibody responses in monkeys, but the IgG antibody titers were surprisingly found to decline close to the baseline level 1 year later.

**Table 1:** The anti-EV71 IgG response after EV71vac immunization.

Group	IgG titer (GMT)#							
	Pre-immune (0 week)	After 1^st^ vaccination$ (3 weeks)	After 2^nd^ vaccination (6 weeks)	After 3^rd^ vaccination (9 weeks)	12 weeks	26 weeks	39 weeks	56 weeks
High dose	<200	1903±5800	9051±22853	12800±22074*	15222±9600	800±2696	1131±1238	476±640
Low dose	<200	1600±2693	4525±4800	7611±10245	7611±4800	476±1450	400±690	238±150
PBS	<200	<200	<200	<200	<200	<200	<200	<200

# The IgG titer was assayed with plates coated with EV71/E59 (B4 subgenotype) and the geometric mean titer (GMT) ± standard error (SE) was shown.

$ Three groups were immunized three times with 10 µg (high dose; n = 4) or 5 µg (low dose; n = 4) of EV71vac vaccine or PBS (n = 2) formulated with alum.

*There is a significant difference between high and low dose group (*p*<0.05, tested by Two-way ANOVA).

### Neutralizing antibody responses against vaccine strains

It is of interest to investigate whether EV71 specific IgG could neutralize the vaccine strain. The EV71-specific neutralization titer (Nt) in the pre-immune serum of all monkeys was below the detection limit (<8). After first vaccination, both high- and low dose immunized monkeys generated neutralizing antibodies and the GMT was found to be 140 and 154 for the high- and low-dose monkey groups, respectively (Table 2). The Nt in the PBS control group was remained <8. After two boosting doses, the GMT in the high- and low-dose immunized monkey groups were increased to 4083 and 5053, respectively at week 9 (Table 2). The booster dose could enhance the neutralizing antibody responses: the Nt of post-3^rd^ dose > Nt of post-2^nd^ post > Nt of post 1^st^ dose. In contrast to the dose-dependent anti-EV71 IgG responses, the GMT of neutralizing antibody was found to be similar in both high- and low-dose immunized monkey groups. Similar to IgG responses, as shown in Table 2 the EV71-specific neutralization ability was found to decrease quickly after the final immunization and the GMT at week 12 dropped to 1446 and 1640 for the high- and low-dose groups, respectively. The GMT in the high- and low-dose immunized groups 56 weeks after vaccination was found to be 450 and 105, respectively. In Table 2 the neutralizing antibody response in low-dose group was shown to decrease significantly and faster than the high-dose group (*p*<0.01). Although the Nt of each EV71vac immunized macaques had dropped significantly every month after the third vaccinations, 100% (4/4) of high-dose immunized monkeys still had Nt >100 at week 56. In contrast, 50% (2/4) of the low-dose group had Nt >100.

**Table 2:** The neutralizing antibody responses after EV71vac immunization.

Group	Nt titers (GMT)#							
	Pre-immune (0 week)	After 1^st^ vaccination$ (3 weeks)	After 2^nd^ vaccination (6 weeks)	After 3^rd^ vaccination (9 weeks)	12 weeks	26 weeks	39 weeks	56 weeks
High dose	<8	140±123	1737±1187	4084±1697**	1446±1188	1126±504	800±581	451±122
Low dose	<8	154±73	1378±315	5054±1404**	1640±494	290±140	263±140	106±81
PBS	<8	<8	<8	<8	<8	<8	<8	<8

# The neutralization titer (Nt) was assayed with EV71/E59 (B4 subgenotype) and the GMT ± SE was shown.

$ Three groups were immunized three times with 10 µg (high dose; n = 4) or 5 µg (low dose; n = 4) of EV71vac vaccine or PBS (n = 2) formulated with alum.

**There is a significant difference compared to PBS control group (*p*<0.01 by Two-way ANOVA test).

### Specific T-cell responses to EV71

**Figure 1:**
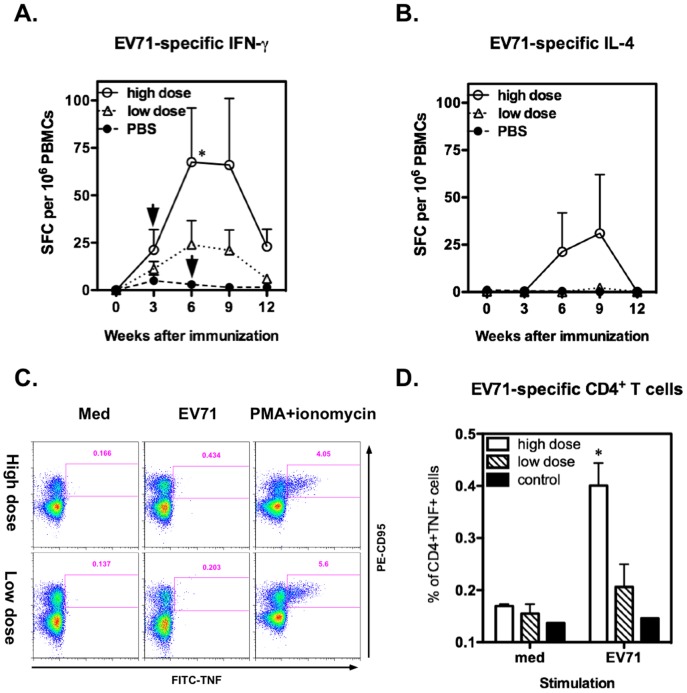
EV71-specific T cell responses induced by EV71vac vaccination. PBMCs from monkeys immunized with high (n = 4) or low doses of EV71vac (n = 4) or PBS control (n = 2) were stimulated with inactivated EV71 virus or medium alone. Specific IFN-γ or IL-4 production responding to EV71 antigen stimulation were assayed by ELISPOT, and the number of spot forming cells per million subtracted from the medium alone is represented (A and B). At week 6, PBMCs from some of the high or low dose EV71vac-immunized monkeys (n = 3) or PBS controls (n = 1) were assayed for the percentage of TNF-producing CD4^+^ T cells by intracellular staining and gated on CD3^+^CD4^+^ population. The typical result for TNF-producing CD4^+^ T cells (CD95^+^ TNF^+^) from one high dose (A31) and one low dose (A11) subject after stimulation with medium, inactivated EV71 or PMA plus ionomycin is shown (C). Data for EV71-specific TNF-producing CD4^+^ T cells from all monkeys are summarized in (D).

To investigate the T cell response, we further measured EV71-specific IFN-γ and IL-4 production by ELISPOT assay. None of the monkeys showed EV71-specific IFN-γ or IL-4 responses before immunization. After immunization, the EV71-specific IFN-γ response was initially detected in one high-dose immunized monkey at week 3 and reached to a peak at week 6 after 1 booster dose (Figure 1A). A dose-dependent IFN-γ response was observed in the EV71vac immunized monkey groups. However, the EV71-specific T-cell responses were found to decline quickly after the final immunization, and only monkeys in the high-dose group retained detectable specific IFN-γ production 6 weeks later. Interestingly, only one high-dose immunized monkey developed EV71-specific IL-4 responses but not the others (Figure 1B). We also performed intracellular staining to measure TNF-producing CD4^+^ T cells in the high- and low-dose EV71vac-immunized monkeys (n = 3) and one PBS control monkey at week 6 (Figure 1C). Without inactivated EV71 virus stimulation, TNF-producing CD4^+^ T cells were an average of 0.159±0.019% of total monkey CD4^+^ T cells. In the stimulation with inactivated EV71 virus, a significant increase TNF-producing CD4^+^ T cells was detected in the high-dose immunized monkeys but not in the low-dose group or PBS control (Figure 1D; *p*<0.05). The EV71-specific TNF-producing CD4^+^ T cells for high dose, low dose and PBS control were shown to be 0.398±0.073, 0.205±0.075 and 0.146, respectively.

### Antigenicity of EV71 capsid proteins

**Figure 2:**
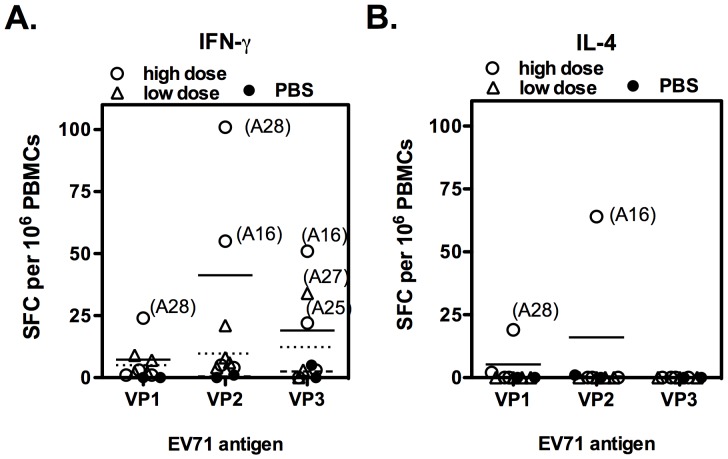
Analysis of dominant T-cell region among EV71 capsid proteins. PBMCs from monkeys that had received a 2^nd^ immunization (week 6) were stimulated with recombinant VP1, VP2 or VP3 protein, and the T-cell response was analyzed by ELISPOT. The IFN-γ (A) and IL-4 (B) response from individual monkeys are represented (blank circle: high dose; blank triangle: low dose; black circle: PBS control), and the mean for high and low dose and PBS control are also indicated by a straight line, dotted line and dashed line, respectively.

To characterize whether EV71vac could induce EV71 capsid protein-specific T-cell immune responses in macaques, the cellular immune responses to different capsid proteins were performed and compared. A series of 57, 49 and 47 overlapping peptides covered VP1, VP2 and VP3 protein were synthesized and used to stimulate EV71-specific T cells [20], [24]. None of the monkeys showed EV71-specific IFN-γ or IL-4 responses before immunization. After the 2^nd^ immunization (week 6), 25% (1/4) of the low-dose immunized group (monkey A27) and 75% (monkeys 16, 25 and 28) of the high-dose immunized group but none of the PBS control group developed specific IFN-γ production (Figure 2A). In contrast, two monkeys (monkey A16 and A28) in the high-dose vaccine group generated specific IL-4 responses (Figure 2B). Compared with different EV71 antigens, VP2 and VP3 were seemed to be the most immunogenic. High level of VP2-specific IFN-γ responses was detected in three different EV71vac immunized monkeys. VP1 was the lowest, and only one monkey (A28) immunized with high-dose vaccine demonstrated weak but significant IFN-γ and IL-4 responses. The EV71-specific IFN-γ response of A28 was induced by VP1 and VP2, but by VP2 and VP3 in macaque A16. The responses of A27 and A25 were induced by VP3. The T-cell antigenicity seems to be caused by individual macaque rather than by viral capsid proteins.

### Cross-neutralizing antibody responses against different EV71 genotypes

It is important to know whether neutralizing antibodies induced by EV71vac could cross-neutralize other EV71 subgenotypes. Eight different EV71 strains and one Coxsackievirus A16 (CVA16) strain were used for the cross-neutralization test. No cross-neutralizing antibody responses (<8) were detected in any pre-immune sera. After immunization, dose-dependent cross-neutralizing antibody responses against EV71-0204 (B1), EV71-0692 (B5), EV71-0448 (B5), EV71-4643 (C2), EV71-C4D (C4a) and EV71-1757 (C4a) were found in EV71vac immunized monkeys and remained detectable even 56 weeks after vaccination (Table 3). In contrast, EV71vac elicited no cross-neutralizing antibody responses against subgenotype C2 (EV71-5746), C4b (EV71-N3340) and CVA16 (CVA16-5079N). These are surprising but interesting results since rabbit and mouse antisera induced by EV71vac in our previous studies [20], [22], [25] could neutralize C2 (EV71-5746) and C4b (EV71-N3340). From our recent clinical trial study [24], about 20% of human sera obtained from adult volunteers immunized with EV71vac could cross-neutralize subgenotype C2 (EV71-5746), C4b (EV71-N3340) and CVA16 (CVA16-5079N). Therefore, this phenomenon seems to be caused by both individual macaque genetic background differences and vaccine strain.

**Table 3:** The cross-neutralization pattern of immunized monkey sera after EV71vac (B4 subgenotype) vaccination.

Virus strain	Subgenotype	After 2^nd^ Vaccination (6 weeks)$		26 weeks		56 weeks	
		High dose	Low dose	High dose	Low dose	High dose	Low dose
EV71-E59	B4	1737±1187#	1378±315	1126±504	290±140	451±122	106±81
EV71-0204	B1	562±65	200±95	71±8	35±4	56±6	28±3
EV71-0692	B5	2021±26	1842±147	750±43	126±29	562±65	119±49
EV71-0448	B5	1059±438	944±275	422±73	127±44	316±36	100±23
EV71-5746	C2	<8	<8	<8	<8	<8	<8
EV71-4643	C2	1122±682	1227±31	841±146	668±38	668±39	473±138
EV71-C4D	C4A	1625±379	202±99	596±103	56±34	398±190	40±30
EV71-1757	C4A	447±272	246±112	422±228	42±29	237±128	30±9
EV71-N3340	C4B	<8	<8	<8	<8	<8	<8
CVA16-5079N	CVA16	<8	<8	<8	<8	<8	<8

# The neutralization titer was assayed with various enteroviruses indicated in the left and the GMT ± SE was presented.

$ Three groups were immunized three times with 10 µg (high dose; n = 4) or 5 µg (low dose; n = 4) of EV71vac vaccine formulated with alum. The PBS control (n = 2) did not show any neutralization activity (<8) to all enteroviruses.

To clarify the difference in cross-neutralization, we also performed a bioinformatics analysis by aligning the amino acid sequence of P1 protein between these strains and the vaccine strain, EV71/E59. The similarity between EV71/E59 and CAV16-5079N was the lowest. The sequence homology between EV71/E59 and other strains such as EV71-4643, EV71-5746, EV71-N3340 and CVA16-5079N was found to be 96.87%, 96.87%, 97.10% and 80.05%, respectively (Figure S1). Even the identity is >96%, there are 27, 27 and 25 amino acid changes between EV71/E59 and EV71-4643, EV71-5746 and EV71-N3340, respectively. These amino acid differences could sufficiently alter the structural and conformational epitope(s) that the neutralizing antibodies would not recognize and prevent viral infections in the neutralization assay.

### Neutralizing antibody responses of monkeys revaccinated again one-year later

To understand the boosting effect of EV71vac-specific memory immunity, at 56 weeks all EV71vac immunized monkeys were revaccinated two times of 5 µg of EV71vac 3 weeks apart. Before the booster dose, the GMT of neutralizing antibody titer was below 500 in all EV71vac immunized monkeys. The neutralizing antibodies increased in all immunized monkeys after the first booster dose and the GMT of Nt were elevated from 450 to 1090 and 106 to 661 for the high- and low-dose groups, respectively (Table 4). Compared to the primary immunized monkeys with a GMT of 140 and 154 in week 3 (Table 2), the quick and strong increase of neutralizing antibodies in the secondary immunized monkeys suggest that a booster response is induced by the existing EV71-specific memory immunity. It is also noted that this memory response was dose-dependent and significant higher neutralizing antibodies were observed in pre-immunized monkeys with the high-dose than those obtained from the low-dose of EV71vac vaccine group (*p*<0.05 by Two-way ANOVA test).

**Table 4:** The neutralizing antibody responses after EV71vac booster one-year later.

EV71vac vaccinated Group	Nt titers (GMT)#			
	Pre-booster (56 weeks)	Booster$	After 1^st^ booster (56+3 weeks)	After 2^nd^ booster (56+6 weeks)
High dose (10 µg)	451±122	EV71vac (5 µg/dose)	1090±137*	1413±67*
Low dose (5 µg)	106±81	EV71vac (5 µg/dose)	661±101	827±121
PBS	<8	PBS	<8	<8

# The neutralization titer (Nt) was assayed with EV71/E59 (B4 subgenotype) and the GMT ± SE was shown.

$ Three groups were boosted two times at week 56 and 59 with 5 µg of EV71vac vaccine for both high dose (n = 4) and low dose (n = 4) group or with PBS formulated with alum (n = 2).

*There is a significant difference compared to low dose group (*p*<0.05 by Two-way ANOVA test).

## Discussion

Several inactivated EV71 whole-virion vaccine candidates have been developed and being tested in human clinical trials [20]–[27]. However, the long-term safety and immunogenicity of these EV71 vaccine candidates is less well reported. Given that EV71 epidemics happen frequently in Asia-Pacific countries [2], [9], it is important for an EV71 vaccine to offer safe and long-term protection. In this study, we reported the long-term (>56 weeks) safety and immunogenicity studies of an inactivated EV71 virus vaccine (EV71vac) in non-human primates (macaques). The current safety data have shown EV71vac immunized macaques to have stable blood count and no local nor systemic adverse effects over the entire 56 weeks study. These results also suggest that a high dose (10 µg) of EV71vac is safe and well tolerated in young macaque model as the safety data shown in the recent human clinical trial [22].

Other EV71 vaccine studies in monkeys [27], [28] were short-term and reported increase in the neutralizing antibody responses, so it was difficult to know their potential for long-term protection. The current results demonstrate that all immunized monkeys after receiving a single EV71vac vaccination regardless to dosage were 100% seroconversion as judged by 4-fold increase IgG antibody responses and had GMT of neutralizing antibody >100. These results are very similar to other monkey immunogenicity reports [27], [28] and our recent clinical studies [22], [24] in which the adult volunteers immunized with 5- and 10-µg dose of EV71vac had shown to have 100% seroconversion rate in their IgG titers and Nt titers. The booster dose could further enhance the neutralizing antibody responses: the Nt of post-3^rd^ dose > Nt of post-2^nd^ post > Nt of post 1^st^ dose. These results are different from the human trial where no significant Nt elicited by the second dose of EV71vac [22]. Although the GMT of neutralizing antibody in vaccinated macaques after 3 immunizations were found to be similar (4084 and 5054 for high and low dose groups, respectively), the decline of Nt generated from 10 µg dose was much slower and found to have higher Nt than those obtained with 5 µg dose at all time point of post-immunization (Table 2). The current results also suggest that 10 µg dose of EV71vac would be better for the long-term immunity since the GMT of neutralizing antibody is found to be >450 from the 56-weeks sera. The similar results were observed with macaques' sera at 33 weeks post immunization with 20 µg dose of inactivated EV71 whole-virion [28].

Although the protective level of neutralizing antibody against EV71 infection in human is unclear, the neutralizing antibodies with Nt >100 have been shown to protect against EV71 infection in mouse challenge model [26]. In the current study, 50% of monkeys after a single dose in both EV71vac immunized groups had generated protective neutralizing antibody responses (Nt >100) at week 3 and all EV71vac immunized monkeys had Nt >100 response after two doses of vaccinations. Although the Nt of each EV71vac immunized macaques had dropped significantly every month after the third vaccinations, 100% (4/4) of high-dose immunized monkeys still had Nt >100 at week 56. In contrast, 50% (2/4) of the low-dose group had Nt >100. To our knowledge, this is the first report of an EV71 vaccine candidate that can induce a year-long protective antibody response against EV71 in a non-human primate model.

The dose-dependent EV71-specific IFN-γ production observed in the immunized monkeys indicated EV71vac induced T-cell responses. Higher T-cell and long-lasting neutralizing antibody responses in the high-dose immunized monkeys suggested that memory T-cell responses might be induced and correlated to the long-lasting neutralizing antibody production. Similar to other monkey immunogenicity study [28], an IFN-γ dominant T cell response was observed in our results. Alum was used as the adjuvant in this study, the immunization with EV71vac did not shift to a bias Th2 response (higher IL-4), but good IFN-γ responses instead. This IFN-γ skewing Th1 response is interesting and unlike other finding with subunit vaccines formulated with alum [29]. The difference may come from the remaining viral RNA in EV71vac that could function as a toll-like receptor agonist as proposed by Lin et al. [28], or the immunogenic nature of EV71 capsid proteins as seen in EV71 infected adults [30]. It has been reported that IFN-γ plays an important role in mice against EV71 [31], [32] and is associated with the reduced severity of HFMD [33]. Therefore, the higher IFN-γ production in EV71vac immunized monkeys should provide strong benefits against EV71 infection. In addition, a dose-dependent T-cell response, including EV71-specific IFN-γ production and a percentage of specific CD4^+^ T cells, was associated with a dose-dependent antibody response. The induced EV71-specific CD4^+^ T cells contribute to the differentiation of memory B cells and maintenance of long-term protective neutralizing antibodies. This is supported by the findings that a dramatic increase (ranging from 2 to 30-fold) in neutralizing antibody responses when the immunized macaques were re-vaccinated again after 56 weeks (Table 4).

Previous EV71 vaccine studies demonstrated that VP1 was the major target for neutralizing antibodies [34], and some CD4^+^ T cell epitopes had been reported in this region [35]. Meanwhile, it has been reported that cross-reactive T cell epitopes for other enteroviruses and poliovirus are mainly located in VP2 and VP3 [36]. Recently, Tan et al. reported that VP2-dominant CD4^+^ T cell responses were detected in EV71 exposed and/or infected humans [30]. Interestingly, our monkey T cell results were correlated to human responses where VP2 and VP3 seemed to be more immunogenic than VP1. High level of VP2- and VP3-specific IFN-γ responses was detected in three different EV71vac immunized monkeys (fig. 2A). VP1 was the lowest, and only one monkey (A28) immunized with high-dose vaccine demonstrated weak but significant IFN-γ response. The individual macaque genetic differences also play important T-cell immune responses. The EV71-specific IFN-γ response of A28 was induced by VP1 and VP2, but in macaque A16 induced by VP2 and VP3. The responses of A27 and A25 were induced by VP3. Given the different regional bias in the epitopes of neutralizing antibodies and CD4^+^ T cells between mice and human [34], [37], together with sharing of a cross-neutralizing pattern and CD4^+^ T cell dominant regions between monkeys and humans in this study, it suggests that monkey is a better animal model than mouse to evaluate immunogenicity of the EV71 vaccine.

Due to the recent epidemics caused by different subgenotypes of EV71, it is important for EV71 vaccine candidates to induce cross-neutralizing antibodies against different genotypes or subgenotypes of EV71. Our B4 subgenotype-based EV71vac elicited cross-neutralizing antibodies to B1, B5 and C4A and partial cross-neutralizing antibodies to C2 but not to C4b and CVA16. It is similar to a human study that demonstrated weak cross-neutralizing antibodies to C2, C4B and CVA16 [22], [24]. The dissimilarity in amino acid sequence may account for the no neutralizing activity to CVA16 (80%), but the sequence homology between B4 and C2 or C4b subgenotypes is found to be >96% (Figure S1). The difference in cross-neutralization between C4a and C4b may result from the genetic substitution or recombination. It has been reported a nucleotide substitution, which could cause an amino acid change in VP1 that had reverted into C4b viruses from C4a [6]. In addition, C4a has been proposed as a recombinant virus of EV71 and CVA16 in the nonstructural protein genes region [6]. But EV71-3340N (C4b) was identified as a recombinant virus between EV71 subgenotype C2 and B2 [7]. The occurrence of intra- and inter-genotypic recombination could cause both genetic and antigenic changes that reduce and prevent the cross-neutralization activity as observed in this study. Between the two C2 subgenotype viruses (EV71-5746 and EV71-4643), we found two amino acid changes (Figure S1) in VP1-145 (Q to E) and VP1-283 (F to S) that might result in the antigenic changes and totally different in the cross-neutralizing activity. With regard to the differences in cross-neutralization patterns and the antigenic determinants among EV71 subgenotypes and other enterovirus such as CVA16 [17], [38], it implies that development of multivalent HFMD vaccines is necessary.

To develop a potent vaccine against EV71, we demonstrate that EV71vac is capable inducing both EV71-specific T-cell and neutralizing antibody responses in macaques; and the protective cross-neutralizing antibodies against different genotypes of EV71 maintain for over one year. Our results here also supported that monkey is a better animal model for EV71 vaccine potency evaluation based on their similarity cross-neutralizing pattern and T cell antigenicity. Therefore, the current monkey results provide a strong rationale for long-term evaluation of EV71vac in phase II human clinical trials.

## Supporting Information

Figure S1 **Sequence alignment and amino acid similarity of the P1protein (1–862 a. a.) between different enteroviruses.** All of the P1 protein sequences were obtained from NCBI protein database and aligned by clustalW alignment. The consensus amino acid sequence was shown in the bottom line and the changed amino acids were indicated. The similarity of sequences was also represented in the bottom table.
(DOC)

Table S1 **Physiological information of 10 monkeys involved in this study and the side effect results after each vaccination.**
(DOC)

Table S2 **The blood count data in monkeys received immunization.**
(DOC)

Checklist S1 **NC3Rs ARRIVE Guidelines Checklist for this study.**
(PDF)

## References

[1] Ho M, Chen ER, Hsu KH, Twu SJ, Chen KT, et al (1999) An epidemic of enterovirus 71 infection in Taiwan. Taiwan Enterovirus Epidemic Working Group. N Engl J Med 341: 929–935. 10498487 10.1056/NEJM199909233411301

[2] McMinn PC (2012) Recent advances in the molecular epidemiology and control of human enterovirus 71 infection. Curr Opin Virol 2: 199–205. 22482716 10.1016/j.coviro.2012.02.009

[3] Lukashev AN, Lashkevich VA, Ivanova OE, Koroleva GA, Hinkkanen AE, et al (2003) Recombination in circulating enteroviruses. J Virol 77: 10423–10431. 12970427 10.1128/JVI.77.19.10423-10431.2003 PMC228507

[4] Kirkegaard K, Baltimore D (1986) The mechanism of RNA recombination in poliovirus. Cell 47: 433–443. 3021340 10.1016/0092-8674(86)90600-8 PMC7133339

[5] Drake JW (1993) Rates of spontaneous mutation among RNA viruses. Proc Natl Acad Sci U S A 90: 4171–4175. 8387212 10.1073/pnas.90.9.4171 PMC46468

[6] Zhang Y, Tan X, Cui A, Mao N, Xu S, et al (2013) Complete genome analysis of the C4 subgenotype strains of enterovirus 71: predominant recombination C4 viruses persistently circulating in China for 14 years. PLoS One 8: e56341. 23441179 10.1371/journal.pone.0056341 PMC3575343

[7] Huang SC, Hsu YW, Wang HC, Huang SW, Kiang D, et al (2008) Appearance of intratypic recombination of enterovirus 71 in Taiwan from 2002 to 2005. Virus Res 131: 250–259. 18036697 10.1016/j.virusres.2007.10.002

[8] Yoke-Fun C, AbuBakar S (2006) Phylogenetic evidence for inter-typic recombination in the emergence of human enterovirus 71 subgenotypes. BMC Microbiol 6: 74. 16939656 10.1186/1471-2180-6-74 PMC1569848

[9] Xu J, Qian Y, Wang S, Serrano JM, Li W, et al (2010) EV71: an emerging infectious disease vaccine target in the Far East? Vaccine 28: 3516–3521. 20304038 10.1016/j.vaccine.2010.03.003

[10] Brown BA, Oberste MS, Alexander JP Jr, Kennett ML, Pallansch MA (1999) Molecular epidemiology and evolution of enterovirus 71 strains isolated from 1970 to 1998. J Virol 73: 9969–9975. 10559310 10.1128/jvi.73.12.9969-9975.1999 PMC113047

[11] Yi L, Lu J, Kung HF, He ML (2011) The virology and developments toward control of human enterovirus 71. Crit Rev Microbiol 37: 313–327. 21651436 10.3109/1040841X.2011.580723

[12] Chang LY, Hsiung CA, Lu CY, Lin TY, Huang FY, et al (2006) Status of cellular rather than humoral immunity is correlated with clinical outcome of enterovirus 71. Pediatr Res 60: 466–471. 16940249 10.1203/01.pdr.0000238247.86041.19 PMC7086547

[13] Lin YW, Chang KC, Kao CM, Chang SP, Tung YY, et al (2009) Lymphocyte and antibody responses reduce enterovirus 71 lethality in mice by decreasing tissue viral loads. J Virol 83: 6477–6483. 19386699 10.1128/JVI.00434-09 PMC2698549

[14] Chang LY, King CC, Hsu KH, Ning HC, Tsao KC, et al (2002) Risk factors of enterovirus 71 infection and associated hand, foot, and mouth disease/herpangina in children during an epidemic in Taiwan. Pediatrics 109: e88. 12042582 10.1542/peds.109.6.e88

[15] Chang LY, Tsao KC, Hsia SH, Shih SR, Huang CG, et al (2004) Transmission and clinical features of enterovirus 71 infections in household contacts in Taiwan. JAMA 291: 222–227. 14722149 10.1001/jama.291.2.222

[16] Foo DG, Alonso S, Chow VT, Poh CL (2007) Passive protection against lethal enterovirus 71 infection in newborn mice by neutralizing antibodies elicited by a synthetic peptide. Microbes Infect 9: 1299–1306. 17890123 10.1016/j.micinf.2007.06.002

[17] Arita M, Nagata N, Iwata N, Ami Y, Suzaki Y, et al (2007) An attenuated strain of enterovirus 71 belonging to genotype a showed a broad spectrum of antigenicity with attenuated neurovirulence in cynomolgus monkeys. J Virol 81: 9386–9395. 17567701 10.1128/JVI.02856-06 PMC1951441

[18] Chen HL, Huang JY, Chu TW, Tsai TC, Hung CM, et al (2008) Expression of VP1 protein in the milk of transgenic mice: a potential oral vaccine protects against enterovirus 71 infection. Vaccine 26: 2882–2889. 18450335 10.1016/j.vaccine.2008.03.041

[19] Chung YC, Huang JH, Lai CW, Sheng HC, Shih SR, et al (2006) Expression, purification and characterization of enterovirus-71 virus-like particles. World J Gastroenterol 12: 921–927. 16521221 10.3748/wjg.v12.i6.921 PMC4066158

[20] Chong P, Hsieh SY, Liu CC, Chou AH, Chang JY, et al (2012) Production of EV71 vaccine candidates. Hum Vaccin Immunother 8: 1775–1783. 22992566 10.4161/hv.21739 PMC3656065

[21] Li YP, Liang ZL, Gao Q, Huang LR, Mao QY, et al (2012) Safety and immunogenicity of a novel human Enterovirus 71 (EV71) vaccine: a randomized, placebo-controlled, double-blind, Phase I clinical trial. Vaccine 30: 3295–3303. 22426327 10.1016/j.vaccine.2012.03.010

[22] Cheng A, Fung CP, Liu CC, Lin YT, Tsai HY, et al (2013) A Phase I, randomized, open-label study to evaluate the safety and immunogenicity of an enterovirus 71 vaccine. Vaccine 31: 2471–2476. 23541623 10.1016/j.vaccine.2013.03.015

[23] Zhu FC, Meng FY, Li JX, Li XL, Mao QY, et al (2013) Efficacy, safety, and immunology of an inactivated alum-adjuvant enterovirus 71 vaccine in children in China: a multicentre, randomised, double-blind, placebo-controlled, phase 3 trial. Lancet 381: 2024–2032. 23726161 10.1016/S0140-6736(13)61049-1

[24] Chou AH, Liu CC, Chang JY, Jiang R, Hsieh YC, et al (2013) Formalin-Inactivated EV71 Vaccine Candidate Induced Cross-Neutralizing Antibody against Subgenotypes B1, B4, B5 and C4A in Adult Volunteers. PLoS One 8: e79783. 24278177 10.1371/journal.pone.0079783 PMC3836818

[25] Chou AH, Liu CC, Chang CP, Guo MS, Hsieh SY, et al (2012) Pilot scale production of highly efficacious and stable enterovirus 71 vaccine candidates. PLoS One 7: e34834. 22529942 10.1371/journal.pone.0034834 PMC3328501

[26] Dong C, Wang J, Liu L, Zhao H, Shi H, et al (2010) Optimized development of a candidate strain of inactivated EV71 vaccine and analysis of its immunogenicity in rhesus monkeys. Hum Vaccin 6: 1028–1037. 21150270 10.4161/hv.6.12.12982

[27] Dong C, Liu L, Zhao H, Wang J, Liao Y, et al (2011) Immunoprotection elicited by an enterovirus type 71 experimental inactivated vaccine in mice and rhesus monkeys. Vaccine 29: 6269–6275. 21722686 10.1016/j.vaccine.2011.06.044

[28] Lin YL, Yu CI, Hu YC, Tsai TJ, Kuo YC, et al (2012) Enterovirus type 71 neutralizing antibodies in the serum of macaque monkeys immunized with EV71 virus-like particles. Vaccine 30: 1305–1312. 22214888 10.1016/j.vaccine.2011.12.081

[29] Chen HW, Liu SJ, Li YS, Liu HH, Tsai JP, et al (2013) A consensus envelope protein domain III can induce neutralizing antibody responses against serotype 2 of dengue virus in non-human primates. Arch Virol 158: 1523–1531. 23456422 10.1007/s00705-013-1639-1

[30] Tan S, Tan X, Sun X, Lu G, Chen CC, et al (2013) VP2 dominated CD4^+^ T cell responses against enterovirus 71 and cross-reactivity against coxsackievirus A16 and polioviruses in a healthy population. J Immunol 191: 1637–1647. 23863902 10.4049/jimmunol.1301439

[31] Shen FH, Tsai CC, Wang LC, Chang KC, Tung YY, et al (2013) Enterovirus 71 infection increases expression of interferon-gamma-inducible protein 10 which protects mice by reducing viral burden in multiple tissues. J Gen Virol 94: 1019–1027. 23288420 10.1099/vir.0.046383-0

[32] Caine EA, Partidos CD, Santangelo JD, Osorio JE (2013) Adaptation of enterovirus 71 to adult interferon deficient mice. PLoS One 8: e59501. 23527208 10.1371/journal.pone.0059501 PMC3602422

[33] Klingel K, Schnorr JJ, Sauter M, Szalay G, Kandolf R (2003) beta2-microglobulin-associated regulation of interferon-gamma and virus-specific immunoglobulin G confer resistance against the development of chronic coxsackievirus myocarditis. Am J Pathol 162: 1709–1720. 12707055 10.1016/s0002-9440(10)64305-2 PMC1851178

[34] Tan CS, Cardosa MJ (2007) High-titred neutralizing antibodies to human enterovirus 71 preferentially bind to the N-terminal portion of the capsid protein VP1. Arch Virol 152: 1069–1073. 17318736 10.1007/s00705-007-0941-1

[35] Kutubuddin M, Simons J, Chow M (1992) Identification of T-helper epitopes in the VP1 capsid protein of poliovirus. J Virol 66: 3042–3047. 1373200 10.1128/jvi.66.5.3042-3047.1992 PMC241064

[36] Cello J, Strannegard O, Svennerholm B (1996) A study of the cellular immune response to enteroviruses in humans: identification of cross-reactive T cell epitopes on the structural proteins of enteroviruses. J Gen Virol 77 (Pt 9): 2097–2108. 8811009 10.1099/0022-1317-77-9-2097

[37] Foo DG, Alonso S, Phoon MC, Ramachandran NP, Chow VT, et al (2007) Identification of neutralizing linear epitopes from the VP1 capsid protein of Enterovirus 71 using synthetic peptides. Virus Res 125: 61–68. 17222936 10.1016/j.virusres.2006.12.005

[38] Huang SW, Hsu YW, Smith DJ, Kiang D, Tsai HP, et al (2009) Reemergence of enterovirus 71 in 2008 in taiwan: dynamics of genetic and antigenic evolution from 1998 to 2008. J Clin Microbiol 47: 3653–3662. 19776232 10.1128/JCM.00630-09 PMC2772620

