# Supplementary material for: Long-Term Immunogenicity Studies of Formalin-Inactivated Enterovirus 71 Whole-Virion Vaccine in Macaques
Source: PLoS One. 2014 Sep 8;9(9):e106756. doi: 10.1371/journal.pone.0106756 (PMC4157806; doi:10.1371/journal.pone.0106756)
Supplement: Figure S1 — Sequence alignment and amino acid similarity of the P1protein (1–862 a. a.) between different enteroviruses. All of the P1 protein sequences were obtained from NCBI protein database and aligned by clustalW alignment. The consensus amino acid sequence was shown in the bottom line and the changed amino acids were indicated. The similarity of sequences was also represented in the bottom table. (DOC) [file pone.0106756.s001.doc]

Figure S1. Sequence alignment and amino acid similarity of the P1protein (1-862) between different enteroviruses.


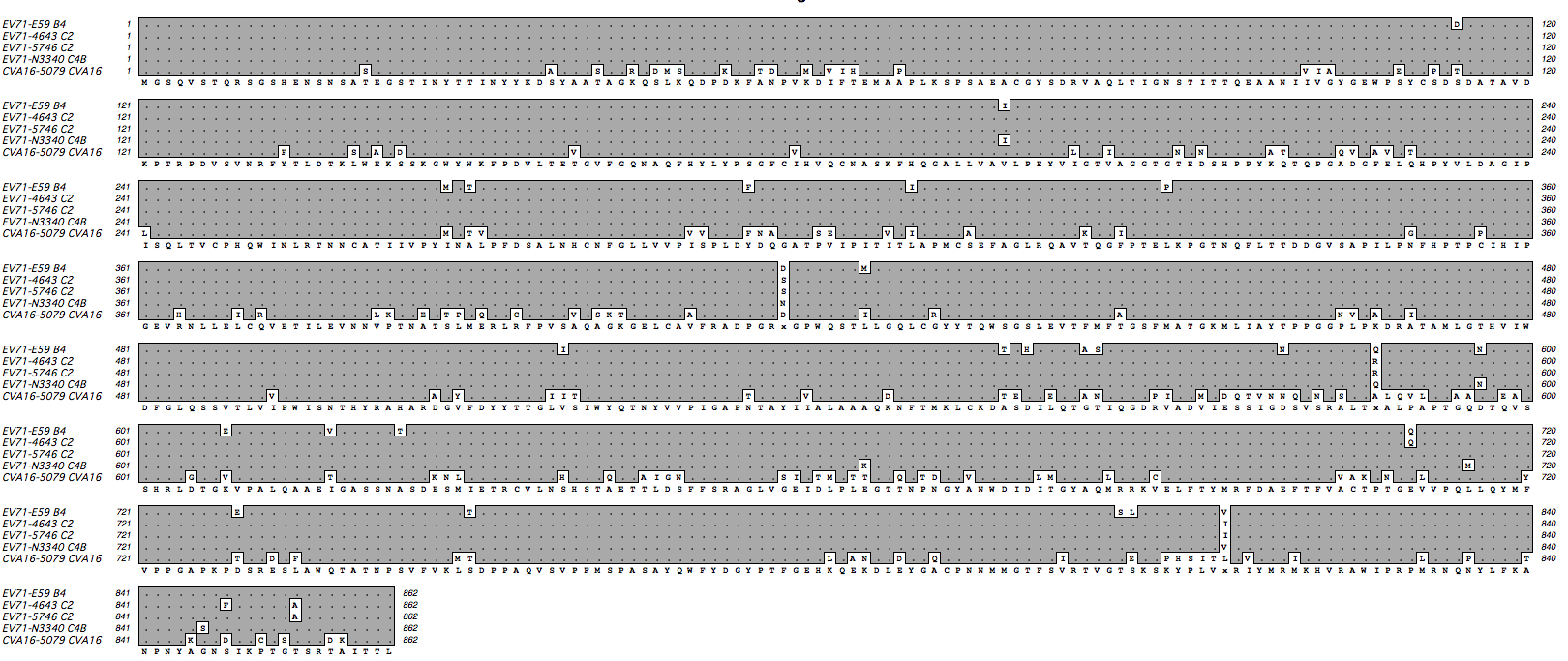


| Similarity | EV71-4643 C2 | EV71-5746 C2 | EV71-N3340 C4B | CVA16-5079 |
| --- | --- | --- | --- | --- |
| EV71/E59 B4 | 96.87% | 96.87% | 97.10% | 80.05% |
