# Supplementary material for: Long-Term Immunogenicity Studies of Formalin-Inactivated Enterovirus 71 Whole-Virion Vaccine in Macaques
Source: PLoS One. 2014 Sep 8;9(9):e106756. doi: 10.1371/journal.pone.0106756 (PMC4157806; doi:10.1371/journal.pone.0106756)
Supplement: Table S1 — Physiological information of 10 monkeys involved in this study and the side effect results after each vaccination. (DOC) [file pone.0106756.s002.doc]

Table S1. Physiological information of 10 monkeys involved in this study and the side effect results after each vaccination.

|  | Group | Gender | Body weight (kg) at beginning | Fever | Redness | Swelling |
| --- | --- | --- | --- | --- | --- | --- |
| A11 | Low dose | M | 2.9 | (-) | (-) | (-) |
| A14 | Low dose | M | 2.5 | (-) | (-) | (-) |
| A16 | High dose | F | 3.8 | (-) | (-) | (-) |
| A18 | Low dose | F | 2.7 | (-) | (-) | (-) |
| A20 | PBS | M | 2.1 | (-) | (-) | (-) |
| A21 | PBS | F | 3.1 | (-) | (-) | (-) |
| A25 | High dose | F | 4.5 | (-) | (-) | (-) |
| A27 | Low dose | F | 1.7 | (-) | (-) | (-) |
| A28 | High dose | M | 5.0 | (-) | (-) | (-) |
| A31 | High dose | M | 1.7 | (-) | (-) | (-) |
