# Supplementary material for: Long-Term Immunogenicity Studies of Formalin-Inactivated Enterovirus 71 Whole-Virion Vaccine in Macaques
Source: PLoS One. 2014 Sep 8;9(9):e106756. doi: 10.1371/journal.pone.0106756 (PMC4157806; doi:10.1371/journal.pone.0106756)
Supplement: Table S2 — The blood count data in monkeys received immunization. (DOC) [file pone.0106756.s003.doc]

Table S2. The blood count data in monkeys received immunization.

|  | RBC^#^ (x10^6^/μl) | | | | WBC (x10^3^/μl) | | | | | PLT (x10^3^/μl) | | | | EOS (%) | | | | |
| --- | --- | --- | --- | --- | --- | --- | --- | --- | --- | --- | --- | --- | --- | --- | --- | --- | --- | --- |
|  | W0^†^ | W6 | W12 | W56 | W0 | W6 | W12 | W56 | W0 | | W6 | W12 | W56 | | W0 | W6 | W12 | W56 |
| A11^$^ | 4.32 | 4.49 | 4.65 | 4.37 | 4.4 | 4.7 | 11.6 | 5.8 | 471 | | 350 | 359 | 401 | | 1.4 | 0 | 0.1 | 0.7 |
| A14 | 4.36 | 4.46 | 4.72 | 4.54 | 7.4 | 6 | 7.1 | 8.1 | 384 | | 364 | 371 | 379 | | 7.3 | 0.9 | 0.5 | 0.3 |
| A16 | 4.28 | 4.23 | 4.21 | 4.59 | 5 | 4.5 | 6 | 5.9 | 395 | | 434 | 444 | 524 | | 1.4 | 3.3 | 3.6 | 0.9 |
| A18 | 4.59 | 4.35 | 4.82 | 4.85 | 3.5 | 4.9 | 4.2 | 6.6 | 390 | | 357 | 378 | 342 | | 2.3 | 2.7 | 5.9 | 2.4 |
| A20 | 4.47 | 4.31 | 4.18 | 4.4 | 14.7 | 9.1 | 6.3 | 6.7 | 319 | | 359 | 186 | 272 | | 0 | 0.8 | 0.7 | 0.8 |
| A21 | 4.53 | 4.55 | 4.72 | 4.48 | 18.3 | 12.3 | 9.1 | 5.4 | 344 | | 320 | 125 | 286 | | 0 | 0 | 0.1 | 0 |
| A25 | 4.52 | 4.58 | 4.54 | 4.4 | 6.1 | 7.6 | 5.8 | 5.8 | 302 | | 440 | 285 | n.d. | | 0.4 | 0 | 0.1 | 0.6 |
| A27 | 4.29 | 4.31 | 4.44 | 4.19 | 4.1 | 5.6 | 4.2 | 4.6 | 288 | | 261 | 264 | 219 | | 4.7 | 7.3 | 6.5 | 2.5 |
| A28 | 3.96 | 3.99 | 4.26 | 4.35 | 4 | 2.9 | 4.9 | 2.7 | 137 | | 229 | 247 | 278 | | 3 | 1.1 | 1.1 | 1.7 |
| A31 | 4.66 | 4.69 | 4.84 | 4.64 | 8.8 | 6 | 6 | 9.8 | 388 | | 398 | 348 | 325 | | 0.8 | 2.5 | 1.7 | 0.5 |

^#^ Abbreviation: RBC: red blood cells; WBC: white blood cells; PLT: platelets; EOS: eosinophils.

^$^ Monkeys were immunized with high dose (A16, A25, A28 and A31) or low dose of EV71vac (A11, A14, A18 and A25) or PBS control (A20 A21) at week 0, 3 and 6.

^†^ Data from pre-immuned (W0), post-2^nd^ vaccinated (W6), post-3^rd^ vaccinated (W12) and the last (W56) monkey blood samples were presented.
